# Trade-off hacking: Have the cake and eat it too? Turning competing interests into win-wins in data-driven food technology

**DOI:** 10.12688/openreseurope.21771.2

**Published:** 2026-02-24

**Authors:** Ferdinand Ferroli

**Affiliations:** 1 Identity Valley Research, Unkel, 53572, Germany

**Keywords:** digital ethics, responsible innovation, privacy, cybersecurity, software development, personalisation

## Abstract

Data-driven innovations in the food sector, from personalised nutrition to supply chain tracking, promise clear benefits but introduce complex trade-offs between competing interests such as personalisation and privacy, or performance and explainability. These tensions can hinder responsible innovation if addressed as zero-sum conflicts. This paper introduces the concept of “Trade-off Hacking”, a user-centric technology design approach that reframes competing interests as opportunities for innovation striving for win-win outcomes. The concept is used as an analytical device to examine the practices of eight pilot projects funded by the Horizon Europe project “DRG4FOOD”. This study applies an exploratory multiple-case study design to eight DRG4FOOD pilots selected through two Horizon Europe open calls (164 admissible proposals; 8 funded). Data sources included pilot applications, implementation documents, and project-level monitoring material generated during the incubation period. Analysis followed two steps: cross-case identification of recurring trade-offs; and pattern identification of resolution strategies into two emerging categories. Trough analysis of these real-world food tech pilots, this study moves beyond merely acknowledging trade-offs to identifying reproducible design patterns that rebalance, or even resolve them. The analysis focuses on three trade-offs (privacy/personalisation, performance/explainability, security/user experience) because these were the most recurrent and comparable across pilots; other tensions are discussed as out-of-scope for the primary framework. The analysis reveals a spectrum of strategies to achieve a rebalancing or resolving, from governance-based user controls and privacy-preserving architectures to co-design methodologies. The paper groups these solutions into two main categories: technology-driven resolutions, which use e.g. architectural or cryptographic methods to influence a trade-off, and cooperation-driven resolutions, which reframe value tensions as socio-technical negotiations.

## Introduction

Digital technology is increasingly reshaping not only the global food system but also the relationship towards the food sector. Innovations ranging from AI-driven personalised nutrition applications to blockchain-based traceability systems offer the potential for promoting public health, empowering consumers, and promoting sustainability (see Ellahi *et al.*, 2023; Wu *et al.*, 2025). However, the integration of data-driven technologies into this critical sector also introduces important ethical and practical conflicts. An application that personalises meal plans based on sensitive health data must simultaneously protect user privacy; an algorithm that predicts the carbon footprint of food products with great precision may not be trusted by retailers and regulators if its calculations are not transparent. These tensions are not merely technical hurdles but represent value conflicts at the core of responsible innovation ( Detopoulou *et al.*, 2023; Donovan *et al.*, 2025; Reis *et al.*, 2021).

Addressing these challenges is a central mission of the Horizon Europe “Digital Responsibility Goals for Food” (DRG4FOOD) project. With a total funding of €4 million, DRG4FOOD aims to foster a data-driven food system that inspires trust throughout the digitalised food chain. The project functions as a responsible technology incubator, having distributed €1.9 million through two Open Calls to eight pilot consortia developing data-driven food tech applications (DRG4FOOD, n.d.). Condition for pilot consortia applying to the incubator programme was the implementation of/adherence to the project’s guiding framework of the seven Digital Responsibility Goals (DRGs). These principles – including Digital Literacy, Cybersecurity, Privacy, Data Fairness, Trustworthy Algorithms, Transparency and Human Agency & Identity – provided the normative backbone for the pilot projects ( Identity Valley, 2025; Meier *et al.*, 2022). This governance structure enabled a real-world laboratory where diverse teams were explicitly tasked with navigating the inherent tensions of building responsible technology while competing under real-world market pressures.

The DRG4FOOD programme design and coaching likely influenced project choices, however, this study does not infer direct causality between incubator support and observed design outcomes. By establishing digital responsibility as a key selection criterion for pilots and providing ongoing coaching and resources, the project aimed to create a distinct ecosystem conducive to responsible innovation ( Ferroli *et al.*, 2023). This environment incentivised and enabled teams to “invest” in resolving complex trade-offs, rather than defaulting to the path of least resistance often dictated by requirements of the market alone. The advanced solutions documented in the pilots – from cryptographic protocols of the consortium “PINACLE” to the comprehensive open-source governance of “Nutrisight” – suggest that the programme’s structure catalysed engagement with responsible design principles ( Ferroli & Weich, 2025). In this context, this “incubator effect” helps understand the successful pilots not as isolated instances, but as potential outcomes of a deliberately created environment for responsible innovation.

It follows that by analysing the practical strategies of the DRG4FOOD pilots through the conceptual lens of “trade-off hacking” we can identify replicable patterns that can help resolve competing interests in other contexts. The evidence from these eight pilot cases suggests that ethical and functional requirements can be designed to be mutually reinforcing rather than oppositional. While achieving a perfect win-win for every stakeholder in every scenario remains a challenging ideal, this analysis moves beyond merely acknowledging trade-offs to conceptualising a constructive empirically grounded path forward for the development of trustworthy and innovative technology for the food sector and beyond.

This paper treats DRG4FOOD as a bounded empirical setting in which teams operated under shared responsibility criteria, not as proof that one funding model universally causes better outcomes. The objective is to analyse how teams navigated value tensions under these conditions and to assess which patterns may transfer to other settings.

## Background

To systematically analyse the paths taken by the DRG4FOOD pilots, this paper proposes the concept of “trade-off hacking”. This section defines the term, situates it within academic discourse, and outlines the analytical framework used in the subsequent analysis.

### Defining “trade-off hacking”

Trade-off hacking is a user-centric approach to digital technology development that reframes a seeming either-or dilemma into an opportunity for innovation. It challenges the assumption that certain design goals are mutually exclusive. Rather than accepting a compromise that weakens both sides of a dilemma – for instance, moderately good personalisation with moderately weak privacy – trade-off hacking seeks to reconfigure the problem space to satisfy both competing interests as much as possible. This approach transforms a zero-sum conflict into a positive-sum innovation challenge. While a perfect positive-sum outcome for all stakeholders (e.g. users, developers, and businesses) may be not achievable, this approach focuses on reconfiguring the problem to move beyond simple compromise and facilitate new, more optimal solutions.

Hence, in this paper, trade-off hacking does not imply that all conflicts are fully resolvable. Some tensions remain structurally persistent, and improvements on one dimension can introduce costs in latency, usability, implementation burden, or governance. A successful “hack” is one that improves outcomes for the most relevant stakeholders in a specific context, without claiming to always benefitting everyone equally.

### Situating the concept in academic discourse

The concept of trade-off hacking is inspired by and builds upon several established theoretical frameworks in technology design and digital ethics.

The concept is an attempt at a practical application of Value-Sensitive-Design (VSD), a methodology that advocates for the proactive and principled integration of human values into the entire technology design process ( Friedman *et al.*, 2008). The dilemmas faced by the DRG4FOOD pilots – as with many product designers and software developers – are conflicts between competing values, such as the value of privacy versus the value of convenience, or transparency versus security. VSD provides the more theoretical frame to address these values explicitly, and trade-off hacking offers a more pragmatic strategy.

Furthermore, the principles of trade-off hacking are also reflected in the emerging field of Value-based Engineering (VBE). Building on the foundational work of VSD, VBE offers a more structured and formalised methodology for integrating ethical values into system design, grounded in the IEEE 7000 standard. Unlike VSD, which provides a broader theoretical framework, VBE focuses on a traceable, step-by-step process that translates identified stakeholder values into concrete technical and organisational requirements, known as Ethical Value Requirements (EVRs) ( Spiekermann & Winkler, 2022). By drawing from VSD, co-design, and software engineering, VBE provides a practical pathway for ensuring that ethical considerations are not just discussed but are systematically embedded and verified throughout the development life cycle.

Aspects of trade-off hacking are also intertwined with participatory and co-design methodologies. These approaches involve stakeholders, particularly end-users, as active partners in the design process ( Sanders & Stappers, 2008). As evidenced by several DRG4FOOD pilots, engaging users and domain experts directly is a powerful strategy for identifying and resolving value tensions. Stakeholders become allies in innovation, offering insights that can reveal novel, win-win solutions that a purely technical/product team might overlook. This transforms the resolution of trade-offs from a top-down technical decision into a collaborative, socio-technical negotiation ( Sadek & Mougenot, 2024; Steinke *et al.*, 2021).

At the foundation of the DRG4FOOD approach stand the Digital Responsibility Goals (DRGs), a framework that reframes society’s relationship with technology by linking digital innovation to human values and trust ( Meier *et al.*, 2022). Much like the United Nations’ Sustainable Development Goals (SDGs) united global actors around a shared sustainability agenda, the DRGs provide a common orientation for a trustworthy digital transformation. Comprising seven interrelated goals – Digital Literacy, Cybersecurity, Privacy, Data Fairness, Trustworthy Algorithms, Transparency, and Human Agency & Identity – they serve as measurable reference points for responsible digital behaviour. In DRG4FOOD, this framework functioned as both governance scaffold and design compass, enabling project teams to translate ethical imperatives into concrete design requirements. The DRGs thus do not merely define abstract ideals but offer a normative structure through which tensions such as privacy versus personalisation or performance versus explainability can be more systematically addressed.

### Establishing the analytical framework of trade-offs

The analysis in this paper is structured around three key trade-offs that are recurrent in the literature and were prominently featured in the experiences of the DRG4FOOD pilots. These three dilemmas serve as thematic pillars for the multi-case analysis:

**Privacy vs. Personalisation:** The tension between the need for user data to deliver tailored services and the imperative to protect users’ sensitive personal information (see e.g. Awad & Krishnan, 2006; Cloarec, 2020).

**Accuracy/Explainability vs. Performance:** The conflict where the most accurate and powerful algorithmic/AI models, such as those based on deep neural networks, are often the most opaque and difficult to interpret, creating barriers to trust and accountability ( Crook *et al.*, 2023; Van Der Veer *et al.*, 2021).

**Security vs. User Experience:** The friction where essential security and compliance measures, such as complex authentication or lengthy consent forms, can create a cumbersome and frustrating user experience, even leading users to circumvent them ( Jacobs & McDaniel, 2022).

These three key trade-offs were selected using three criteria: recurrence across pilots, direct relevance to DRG-aligned design decisions, and sufficient evidence for cross-case comparison. Other tensions (e.g., cost vs. inclusivity, or speed-to-market vs. participatory design) were observed but occurred less consistently or lacked enough comparable evidence across all pilots. This framing improves analytical focus, however, also narrows the generalisability of conclusions.

## Methodological approach

This study adopts an Exploratory Case Study approach (cf. Mills et al., 2010, 372f ). An exploratory design is appropriate because “trade-off hacking” is still conceptually emergent and the goal is analytic clarification and pattern identification rather than hypothesis testing. Cases were included if they developed a data-driven food technology, engaged at least one DRG-relevant value tension, and provided sufficient documentary evidence for cross-case analysis. Data sources included pilot applications, implementation documents, and project-level monitoring material generated during the incubation period. For each case, trade-offs, implemented mitigation strategy and enabling conditions were identified. Then patterns were matched across cases to classify strategies as technology-driven or governance-driven.

## Trade-off hacking: vignettes from the DRG4FOOD pilots

The eight pilot projects selected and supported by the DRG4FOOD consortium provide for some valuable “micro” case studies to observe how trade-offs are navigated in practice. The DRG4FOOD open call selection process for the pilot projects asked applicants to specify in their proposal not only the market viability and scalability of their solutions but decidedly also their measures to achieve digital responsibility (as defined by the DRGs, see Identity Valley, 2025; Meier *et al.*, 2022). In total 164 proposals between two calls were admissible with an average score of 3,6 (call #1) and 3,7 (call #2) out of five for criteria of digital responsibility ( Ferroli & Weich, 2025). This additional requirement for digital responsibility was well-received and understood by applicants, nudging them into a situation where they needed to confront design conflicts already from ideation and navigate trade-offs they otherwise might not have.

The following micro case studies, or vignettes, are not presented as fundamental innovations in responsible technology but rather as small-scale, practical examples to underpin the argument that the deliberate engagement with design tensions yields replicable strategies to resolve them. The pilots exhibit progress in rebalancing these trade-offs, even if they cannot completely resolve them. A comparative analysis of strategies organised by the key trade-offs identified will be presented in this section. Table 1 provides a high-level overview of the pilots and the primary trade-offs they faced and addressed.

**Table 1.** Overview of DRG4FOOD Pilot Projects and Key Trade-Offs.

Project Name	Core Functionality	Key Trade-Off
**ATTESTED**	Farm-to-fork traceability system for small producers using IoT, RFID, and portable sensors.	Security vs. User experience
**Cacao-Tech **	Cacao quality and traceability platform using near-infrared technology and a tracking technology.	Privacy vs. Personalisation (for framers); Security vs User Experience
**DISH**	Personalised recipe application using machine learning and symbolic AI, focusing on user privacy.	Privacy vs. Personalisation; Explainability vs. Performance
**GENIE**	Ultra-personalised nutritional recommender based on genetic, gut microbiota, and blood test data.	Privacy vs. Personalisation
**NutriSight**	AI tool to automatically extract nutritional information from food packaging photos for Open Food Facts.	Explainability vs. Performance
**NutriWell**	AI-based personalised nutrition platform for elderly individuals, with a focus on social inclusion.	Privacy vs. Personalisation; Explainability vs. Performance
**PINACLE**	AI-driven nutrition recommender matching donated food with recipients’ dietary needs using blockchain.	Privacy vs. Personalisation
**SafeNutriKids**	AI-driven personalised nutrition education app for children aged 6–12.	Security vs. User Experience; Privacy vs. Personalisation

### Reconciling personalisation and privacy: from data collection to user sovereignty

The “personalisation-privacy-paradox” describes the tension where services need (sensitive) personal data to be effective, yet users are wary of sharing it due to privacy concerns ( Chellappa & Sin, 2005; Xu *et al.*, 2011). The DRG4FOOD pilots showcase a spectrum of strategies to resolve this dilemma, moving beyond simple consent models toward true user sovereignty. These solutions can be categorised along a continuum of intervention, from governance-based controls to fundamental architectural and cryptographic redesigns.

At the governance and user interface level, the GENIE project provides a suitable example. Tasked with handling exceptionally sensitive genetic data the team implemented a multi-layered approach. The core strategy was to combine standard backend measures, such as anonymising user data and separating personal information from the analytical dataset, with a user-facing “data control panel”. This panel empowers users to actively manage their preferences in detail and opt out of data sharing at any time, shifting the dynamic from passive consent to active control.

Moving to the architectural level, the DISH project demonstrates a different approach. To deliver personalised recipes, the team made deliberate architectural choice to store user profiles and persona information “on-device” only. By redesigning the data flow to avoid centralised collection of personal data, they effectively dissolve the privacy risk at its source. Personalisation is achieved through client-side processing, demonstrating that it is possible to deliver a tailored experience without extracting and aggregating sensitive data on a central server. This approach makes the trade-off less about balancing risks and more about eliminating them through system design.

A more technologically focused approach is found in the PINACLE project, which leverages cryptographic methods to make the trade-off obsolete. PINACLE, which matches donated food with recipients’ needs, uses a combination of Self-Sovereign-Identity (SSI) principles, Verifiable Credentials (VCs), and Zero-Knowledge-Proofs (ZKPs). Zero-Knowledge-Proofs are a cryptographic protocol that allows one party to prove to another that a statement is true without revealing any information beyond the validity of the statement itself ( Garimella & Conway, 2024). In PINACLE’s context, this allows a food recipient to prove eligibility without disclosing sensitive information. This represents a significant shift: the conflict between verification (needed for personalisation/matching) and privacy is not balanced but is rendered virtually irrelevant. The system can verify a user’s dietary needs or status without ever “seeing” the underlying sensitive data. Paired with VCs, which give users granular, portable control over their own data, this approach enables true user sovereignty ( Sedlmeir *et al.*, 2021).

Together, these three cases illustrate that resolving the privacy dilemma is not a monolithic strategy. It exists on a spectrum: GENIE’s approach is about governing data flows, DISH’s is about re-structuring them, and PINACLE’s is about cryptographially transforming the nature of verification itself. This provides a valuable framework for practitioners, who can select the level of intervention best suited to their context, resources, and the sensitivity of the data they handle.

Yet, while several pilots improved privacy protections, an open question is whether stronger safeguards may, in some contexts, reduce data availability for ongoing model improvement. Similarly, privacy-enhancing approaches (e.g., advanced cryptographic methods) may introduce integration demands not yet observable. This suggests the trade-off may be, in many cases, reconfigured rather than removed, with possible costs shifting from privacy risk toward technical and organisational effort.

### Explainability and performance: Building trust in a black box

A central challenge in modern AI is that the most powerful predictive/generative modesl, particularly in deep learning, often function as “black boxes”, making their “reasoning” opaque ( Hassija *et al.*, 2023). This lack of transparency can be a major barrier to user trust and adoption, especially in important domains like health and nutrition. The pilots developed several strategies to provide explainability – and for that matter ensure accuracy – without impinging on performance, by, for example, resorting to less efficient algorithms.

The NutriWell project, which generates personalised nutrition plans for the elderly, demonstrates a proactive, user-centric approach to explainability. Their platform features “Info Points” strategically placed throughout the interface. These provide detailed, contextual explanations of the AI’s recommendations, including insights into their sources, and the scientific foundations behind them, along with the direct links to relevant scientific publications. Furthermore, the platform visualises the precision of its AI by showing users the percentage deviation between the generated meal plan and their initial nutritional requirements. This approach builds trust not by simplifying the underlying model, but by making its outputs transparent, verifiable, and comprehensible to the user.

A different strategy is exhibited by NutriSight, which uses a neural model to extract nutritional data from images. The team resolved the explainability dilemma by implementing a robust “human-in-the-loop” validation system. Their setup ensures that every prediction is validated by a contributor before it is integrated into the Open Food Facts database. This is a pragmatic solution: It allows the project to leverage a high-performance, complex model, while relying on human oversight as the guarantor of accuracy and trustworthiness. Here, trust is not established through an explanation of the algorithm’s internal workings but through the human verification process itself.

Finally, the DISH project chose an approach based on technology selection. The team uses methods from the domain of symbolic AI that allows reliable tracing of the computation process back to the original information sources. Unlike deep learning models, symbolic AI systems operate on more explicit rules and logic, making their “reasoning” inherently transparent and traceable ( Attoresi, 2025; Yu *et al.*, 2023). In this case, the trade-off is addressed at the foundational level by selecting an AI paradigm where explainability is a native property. This approach prioritises transparency, potentially trading a degree of predictive performance for comprehensibility.

A further issue to examine is whether explainability gains could create operational constraints under real-world workload conditions. For example, human-in-the-loop validation may improve trust but could become a bottleneck as deployments scale. Future evaluations should therefore test whether these approaches deliver stable resolutions over time, rather than one-off improvements.

### Integrating security and user experience

Security and regulatory compliance are essential for any digital service, but their implementation often introduces friction that can negatively impact user experience, leading to frustration or non-compliance ( Lennartsson *et al.*, 2021). The pilots show that by treating security not as a technical checklist but as a user-centred design challenge from the beginning, it is possible to create systems that are both safe and seamless.

The SafeNutriKids project faced a version of this dilemma, as it was designed for children, a vulnerable user group requiring high standards of data protection and parental consent. Their solution was to reframe these stringent compliance requirements as an opportunity for user-centric design. Instead of presenting parents with legalistic consent forms, the app communicates its data policy to children through age-appropriate, interactive explanations, using visual cues and simple language. This approach transforms a legal necessity into an engaging and educational feature. It enhances security by fostering genuine understanding and informed consent. While simultaneously improving the user experience for both children and parents.

The Cacao-Tech project provides another example: The team’s traceability platform encountered early concerns from cocoa buyers about sharing competitively sensitive data regarding their farmers. A purely technical solution – e.g. simply stating that the data is encrypted – might not have been sufficient to build trust in a relationship-based industry. The team’s solution was therefore more social, committing to a participatory approach of co-designing/co-developing the tools with the users. By actively involving farmers, buyers, and other stakeholders in the design of the data sharing and security mechanisms, they ensure the final system is not only technically secure but also accepted and usable within the real-world context of the cacao industry. This co-design process pre-emptively alleviates the security-UX trade-off by ensuring the security model is one that users themselves have helped create, validate, and therefore feel ownership over.

However, while co-designing with users can build trust and credibility, it can also take significant time and facilitation capacity, which may be difficult for teams with limited resources. This means that, in practice, teams may need to balance the depth of participation with what is realistically feasible in their operational context.

## Synthesis: Two patterns of trade-off hacking

The insights from the DRG4FOOD pilots allow for a synthesis that groups their practices into two overarching types of patterns for the resolution of conflicts: technology-driven resolutions and governance-driven resolutions. This framework helps classify the strategies used to transform design conflicts towards win-win outcomes, or at least into significantly more favourable trade-offs than traditional compromises. While a more detailed categorisation could have been made, it became clear that the success of many resolutions is tied to many more factors which are specific to the application/use case at hand. In the light of this, the synthesis below – summarised in Table 2 – can be seen as a rough charting of the territory rather than a guideline for exact reference.

**Table 2.** Overview of Trade-Off Hacking Patterns in DRG4FOOD Pilots.

Project Name	Key Trade-off	Resolution	Pattern Category
**DISH**	Privacy vs. Personalisation/ Explainability vs. Performance	On-device data storage and processing/Use of inherently transparent Symbolic AI	Technology-driven
**PINACLE**	Privacy vs. Personalisation	Zero-Knowledge Proofs (ZKPs) for verification	Technology-driven
**GENIE**	Privacy vs. Personalisation	User-facing data control panel for granular consent	Governance-driven
**NutriWell**	Explainability vs. Performance	"Info Points" in UI to explain AI recommendations	Governance-driven
**SafeNutriKids**	Security vs. User Experience	Interactive, age-appropriate consent mechanisms	Governance-driven
**NutriSight**	Explainability vs. Performance	Human-in-the-loop validation of all AI outputs	Governance-driven
**Cacao-Tech **	Security vs. User Experience	Co-design of data sharing rules with stakeholders	Governance-driven

### Technology-driven hacks

This category of patterns involves embedding the solution to conflicting interests directly into the technical setup of the system. These approaches rethink the underlying architecture, algorithms, or cryptographic protocols to eliminate the trade-off at its source. For example, the DISH project tackled the personalisation-privacy paradox by making fundamental architectural choice to store and process all user data exclusively on the user’s device. This design dissolves the privacy risk of data transfers and storage, demonstrating that a tailored user experience does not require a sacrifice of personal data.

Taking this principle a step further, the PINACLE project employed advanced cryptographic methods like Zero-Knowledge-Proofs (ZKPs). This technology allows the system to verify a user’s eligibility for a food donation without ever needing to see the sensitive personal data that proves eligibility, rendering the conflict between verification and privacy risks obsolete. The choice of algorithm itself can also be a successful resolution. DISH again provides an example by opting for symbolic AI, whose rules-based nature makes its “reasoning” inherently transparent. This choice prioritises explainability, addressing the “black box” problem before it arises, rather than trying to explain an opaque model after the fact. In each case, the resolution is not a feature added on top of the system, but it *is* the system.

### Governance-driven hacks

This category reframes value tensions as socio-technical challenges that are best solves through better rules, processes, user controls, and stakeholder collaboration. These solutions build trust not just through code, but though transparency, agency, and shared ownership of how the technology operates. A common pattern is empowering users with direct control. The GENIE project, handling extremely sensitive genetic and health data, implemented a user-facing “data control panel” that allows individuals to actively manage their sharing preferences, turning passive consent into active data governance. This focus on clarity and agency is also visible in how pilots approached the provision of complex information. Instead of dense legal text, SafeNutriKids transformed consent forms into age-appropriate, interactive explanations, making compliance more of an engaging experience. Similarly, NutriWell used “Info Points” within its interface to offer clear, contextual justifications for its AI’s nutritional advice, building user trust through transparency.

Beyond individual user interfaces, governance-driven resolutions can also shape the operational process. The NutriSight project, for instance, uses AI to extract data but govern its use with a strict “human-in-the-loop” protocol, where every automated entry is validated by a person. Here, trustworthiness comes from the process, not the algorithm. The collaborative principle is also present in the Cacao-Tech project, which resolved data-sharing fears among competitors by co-designing the system with its stakeholders. By making the design process itself a “negotiation”, the final technology is shaped with the trust of its stakeholders.

### Limitations

Technology-driven patterns are strongest when risks can be technically specified and teams have advanced engineering capacity. However, they often require higher investment, specialised skills, and longer integration cycles. Governance-driven patterns can be faster to implement and improve legitimacy, but their effectiveness depends on participation quality, competence, and institutional trust. In practice, the most robust cases might be hybrid: governance to define acceptable constraints, followed by targeted technical enforcement.

## Discussion

A central limitation is the “incubator effect”: pilots operated under funding, coaching, and responsibility-oriented selection criteria that are not typical of many commercial environments. Without these supports, teams may prioritise speed-to-market and short-term revenue over resource-intensive responsible-design measures.

Replication outside Horizon Europe is plausible but conditional on enabling factors: dedicated budget for responsible design, increased governance capacity by facilitators of framework programmes for research and development, cross-disciplinary expertise, and stakeholder access. Applicability is likely lower in low-margin markets, low-trust institutional contexts, or organisations with limited technical maturity.

Yet, the analysis of the DRG4FOOD pilots and the emergent patterns of trade-off hacking carry some useful implications for practitioners, policymakers, and the financial institutions funding digital innovation.

### The role of the responsible technology incubator

Above insights suggest that the structure of the DRG4FOOD project itself played a role in fostering these innovative solutions. By making digital responsibility a primary criterion for selection and funding, the program highlighted its importance and created a meaningful incentive. The provision of coaching and resources further provided the capacity for teams, many of whom are SMEs or startups, to invest time and their own resources into solving the value tensions present. This “responsible incubator effect” suggests that funding mechanisms like Horizon Europe can be powerful levers for promoting responsible practices and innovation: To cultivate trade-off hacking, such programs should not only fund technology development but also explicitly reward and support the interdisciplinary and user-centred processes required to resolve value tensions. This de-risks the investment in responsible innovation, which might otherwise be sidelined in favour of more immediate commercial goals.

### Implications for practitioners

For technology developers, the central takeaway is that while trade-offs can rarely be completely resolved to achieve a win-win for all stakeholders, the pursuit of a better resolution is a powerful driver of innovation, and potentially adoption. The “Edelman Trust Barometer Special Report: Tech Sector” series of global surveys (n = 32.000+) have consistently found that trust is tied to adoption. In their 2024 report, for example, they found that the largest barrier to AI adoption are concerns about privacy ( Edelman, 2024, p. 28). However, while a solution like on-device processing is a clear win for user privacy, it may not be a win for a business whose revenue or innovation strategy depends on collecting centralised data. This is exactly what “trade-off hacking” can be valuable as a way of thinking. It nudges practitioners to constantly seek better optima. In the above example, a next-level solution like federated learning might bring the desired win-win, as data stays localised on user devices while the business can still profit from aggregated model insights without infringing on individual privacy ( Loftus *et al.*, 2022). Therefore, the goal is to cultivate this mindset, viewing trade-offs not as constraints but as “innovation prompts”.

The patterns identified in this paper – from user-centric control panels to privacy-preserving cryptography – provide a more nuanced toolkit for responding to these prompts. This requires a dual skillset: the technical know-how to identify and implement technology-driven solutions and the “social skills” to guide governance-driven resolutions through co-design and transparent communication. Adopting an interdisciplinary approach, bringing in expertise from ethics, law, or social sciences in addition to engineering, is most effective for identifying and implementing those more holistic solutions.

Development teams should prioritise technology-driven strategies when data-related risks are high, the technical architecture can be adapted, and there is sufficient long-term capacity to maintain and update solutions. Governance-driven strategies might be more appropriate when values are contested, workflows are highly context-dependent, or rapid legitimacy-building with stakeholders is critical. Under real-world constraints, a hybrid approach might often be most effective: begin with governance (e.g., problem framing, consent design, and accountability arrangements), then introduce targeted technical controls where risks remain. Across contexts, decision-making in these cases could usefully be informed at least by expected scale, implementation cost, organisational maturity, and regulatory exposure.

### Implications for policy-makers

The experiences of the pilots indicate that regulations like the EU General Data Protection Regulation (GDPR), while sometimes perceived as burdensome, can act as powerful catalyst for innovation. The need to comply with stringent privacy and consent requirements pushed teams like SafeNutriKids and PINACLE to develop solutions that not only meet the legal requirements but also enhanced user trust and thereby created a USP and potential competitive advantage. This suggests that policy should not only set high standards for digital responsibility but also actively support the ecosystem needed to meet them. This includes fostering environments where innovators are encouraged and equipped to pursue the ongoing process of optimising trade-offs, funding research into privacy-preserving technologies, promoting the dissemination of successful design patterns, and fostering environments where innovators are encouraged and equipped to find smart, user-centric implementations of regulatory requirements.

### Future research

This investigation is based on an analysis of eight pilot projects within a specific, EU-funded programme. While this provides a coherent dataset, the findings are not generalisable to all contexts and serve primarily as a mental stepping stone to facilitate deeper conversations about re-thinking digital technology development. Future research should include longitudinal studies to track the long-term success and scalability of these “hacked” solutions in the market. Comparative analyses of projects developed outside of a “responsible incubator” context is needed to determine the real extent to which these patterns are adopted organically. Further investigation into the practical implementation of the emergent patterns, particularly the organisational and technical requirements for implementing effective technology- or governance-driven “hacks”, would be a rewarding path for future work. Also, there is a need to develop and apply harmonised outcome indicators across comparable digital solutions so that cross-case analysis can move beyond qualitative pattern mapping and assess relative effectiveness more robustly.

## Conclusion

In the pursuit of a data-driven food system that is sustainable, healthy and fair, it is tempting to view digital technology as a rigid means to an end: that we must choose between personalisation and privacy, performance and transparency or good user experience and security. This paper has argued that this framing often stifles innovation needed to move forward in creating a more user-centric digital ecosystem – and therefore also realise the ideals of a functioning data-driven food system.

Adopting a mindset of “trade-off hacking” – an intentional, creative and human-centred approach to digital technology development – opens new pathways to convert these either-or choices into both-and opportunities.

The eight pilot cases analysed indicate that trade-offs can often be reconfigured, but rarely entirely resolved, and usually at the cost of additional technical, organisational, or governance complexity. Trade-off hacking is therefore best treated as a “constrained design heuristic” rather than a universal recipe for win-win outcomes. Future studies should test these patterns in non-incubator settings and report failed repliactions as systematically as successful adaptations.

A useful contribution of this analysis is the identification of two distinct types of patterns that advance our understanding of how tensions in digital technology development manifest and how their resolutions can be conceptualised. The first, technology-driven resolutions, is about rendering trade-offs obsolete through innovative use of technology. The second, governance-driven, highlights the important role of transparent processes, user agency, and co-design in navigating socio-technical tensions that technology alone cannot solve.

For practitioners and policymakers, the message is: trade-off hacking is not about ignoring difficult choices but about confronting them with a mindset that places human values at the center of innovation. While the perfect win-win is seldom achievable, this approach is fundamental to the continuous process of building trust in digital technology, which is the essential ingredient for any digital transformation to succeed.

## Ethics and consent

Ethical approval and consent were not required for this study.

## Declarations

### Declaration of generative AI and AI-assisted technologies in the writing process

During the preparation of this work the author used ChatGPT (GPT-5) for minor improvements on language and readability. After using the tool, the author reviewed and edited the content and takes full responsibility for the content of the published article.

## Data Availability

No data associated are with the article. No datasets were generated or analyzed during the preparation of this case study.
