# Supplementary material for: Trade-off hacking: Have the cake and eat it too? Turning competing interests into win-wins in data-driven food technology
Source: Open Res Eur. 2026 Feb 24;5:358. Originally published 2025 Nov 24. [Version 2] doi: 10.12688/openreseurope.21771.2 (PMC12988359; doi:10.12688/openreseurope.21771.2)
Supplement: Peer review history

# Reviewer report: Reviewer response for version 2

**DOI:** 10.21956/openreseurope.25084.r70356

**Published:** 2026-03-13

**Authors:** Agnieszka Kurdyś-Kujawska

**Affiliations:** 1 Koszalin University of Technology, Kwiatkowskiego, Koszalin, Poland

**General comments:** The article is a coherent and valuable contribution to the discussion on digital ethics in the agri-food sector. It effectively combines a high level of theory with practical examples of implementation, making it useful for both researchers and software developers. A strong point of the study is its departure from theorizing about ethics in favor of showing how values can become the basis for technical creativity.

**Abstract: **The abstract is complete and contains all the key elements required of a scientific abstract.

**Introduction:** The introduction is well structured, with the author moving smoothly from the broad technological context to specific ethical dilemmas, and then to a concrete methodology and analytical framework.

**Background:** The background to the article is a well-constructed analytical tool that allows the reader to understand why and how the pilot projects studied attempted to overcome traditional technological limitations.

**Hacking compromises: excerpts from the DRG4FOOD pilot:** The section is structured around three key dilemmas, allowing for a clear comparison of different approaches to the same problem. The author does not limit himself to describing the projects, but uses them as “micro-case studies” to illustrate specific strategies. The selection of eight pilots ensures adequate technological diversity – from mobile applications to blockchain systems in the supply chain. The text accurately identifies the spectrum of interventions, which is one of the strongest points of this section. The author maintains the necessary objectivity, avoiding claims that all dilemmas have been completely eliminated. Despite its high analytical value, the text is based mainly on qualitative and narrative descriptions. It lacks hard data or harmonized indicators (metrics) that would allow for an objective comparison of the effectiveness of individual “hacks.” The analysis focuses on the declared strategies from the incubation period, which, without long-term research, does not provide complete certainty as to the ultimate effectiveness of these solutions when used on a large scale.

**Synthesis: Two patterns of trade-off hacking:** The section entitled “Synthesis: Two patterns of trade-off hacking” provides a theoretical and practical summary of the analysis, giving the work a systematic character. Here, the author moves away from describing individual projects in favor of creating a universal framework for classifying strategies for resolving project conflicts. The analysis remains at the qualitative level. Although the author has created a solid theoretical framework, it lacks more precise criteria for choosing between a technological and a managerial pattern depending on, for example, the size of the organization or the degree of technological maturity of the project.

**Discussion: **This section constitutes the analytical layer of the work, going beyond the description of specific cases towards a synthesis of conclusions for the entire food technology sector. Despite the high quality of the text, I see areas that could be explored in greater depth to enhance the scientific value of this section. The discussion remains at a narrative and qualitative level. There is a lack of specific indicators that a developer could use to measure how much better a “hacked” compromise is than a standard solution. The author mentions the risk of “bottlenecks” (e.g., in human verification), but the discussion lacks a broader analysis of how solutions such as human-in-the-loop or co-design will fare in the transition from pilot phase to mass market scale. Although “low-margin markets” are mentioned, the discussion could have analyzed in more detail how small and medium-sized enterprises (SMEs) operating outside EU funds can realistically finance costly technological “hacks” (e.g., ZKP cryptography). The argument is very strongly rooted in the European reality. It would be an improvement to address whether these models are applicable in jurisdictions with different data protection standards or lower trust in institutions.

**Conclusions:** The conclusions section is a logical conclusion to the article. It effectively combines a high level of theory (digital ethics) with practical conclusions for developers and decision-makers, emphasizing that building trust in technology is a continuous and demanding process. Despite its high quality, there are some areas in the conclusions that could be explored in greater depth. The author mentions the costs in terms of complexity, but does not indicate how organizations should assess whether a given “hack” is economically viable in the long term. The conclusions focus on paths to success. There is a lack of emphasis on the boundary conditions that make trade-off hacking doomed to failure, which would be a valuable warning for practitioners.

Is the case presented with sufficient detail to be useful for teaching or other practitioners?

Yes

Is the work clearly and accurately presented and does it cite the current literature?

Yes

If applicable, is the statistical analysis and its interpretation appropriate?

Not applicable

Are all the source data underlying the results available to ensure full reproducibility?

Partly

Are the conclusions drawn adequately supported by the results?

Yes

Is the background of the case’s history and progression described in sufficient detail?

Yes

Reviewer Expertise:

agricultural economics, risk management in agriculture, financing of the agri-food sector

I confirm that I have read this submission and believe that I have an appropriate level of expertise to confirm that it is of an acceptable scientific standard, however I have significant reservations, as outlined above.

# Reviewer report: Reviewer response for version 2

**DOI:** 10.21956/openreseurope.25084.r70274

**Published:** 2026-02-25

**Authors:** Juan Manuel Vargas-Canales

**Affiliations:** 1 Universidad de Guanajuato, Salvatierra, Mexico

 After reviewing the revised version of the manuscript, I can confirm that the previously raised observations have been satisfactorily addressed. The author implemented substantial and appropriate adjustments in each of the highlighted sections, strengthening the conceptual clarity, methodological transparency, and critical balance of the analysis.

 In particular:

- The abstract now explicitly describes the analytical approach used.
- The introduction adopts a more neutral and analytical tone.
- The conceptual framework adequately expands on the notion of *trade-off hacking* and justifies the selection of the three trade-offs.
- The vignette section incorporates additional critical elements, including consideration of tensions that remain unresolved.
- The synthesis section deepens the discussion of limitations associated with the identified patterns.
- The discussion provides a more critical analysis regarding the project context, replicability, and external factors.
- The conclusions have been adjusted to reflect a more cautious and balanced tone.

With these modifications, the manuscript now presents a more robust, critical, and methodologically transparent version, satisfactorily addressing the recommendations previously provided.

Is the case presented with sufficient detail to be useful for teaching or other practitioners?

Partly

Is the work clearly and accurately presented and does it cite the current literature?

Yes

If applicable, is the statistical analysis and its interpretation appropriate?

Not applicable

Are all the source data underlying the results available to ensure full reproducibility?

No source data required

Are the conclusions drawn adequately supported by the results?

Partly

Is the background of the case’s history and progression described in sufficient detail?

Partly

Reviewer Expertise:

Economy, innovation, technological change, digital agriculture

I confirm that I have read this submission and believe that I have an appropriate level of expertise to confirm that it is of an acceptable scientific standard.

# Reviewer report: Reviewer response for version 1

**DOI:** 10.21956/openreseurope.23550.r68250

**Published:** 2026-02-03

**Authors:** Juan Manuel Vargas-Canales

**Affiliations:** 1 Universidad de Guanajuato, Salvatierra, Mexico

**Trade-off hacking: Have the cake and eat it too? Turning competing interests into win-wins in data-driven food technology**

**General comments:**

- The work addresses a real and highly topical problem. It is highly relevant to policy design. However, there are some areas that need improvement.

**Abstract**

- It is not specified how the pilot projects were explicitly analyzed.

**Introduction**

- This section contains statements heavily biased towards justifying the project. It would be preferable for it to be more neutral.

**Background**

- Trade-off hacking: this concept should be expanded upon as much as possible. It would be beneficial to explore the issues further.
- Regarding the trade-offs, it would be helpful to explain why there were only three.

**Trade-off hacking: vignettes from the DRG4FOOD pilots**

- This section would be strengthened if it were more critical. Furthermore, it would be advisable to include failures, unresolved tensions, and aspects not yet considered.

**Synthesis: Two patterns of trade-off hacking**

- In this section, it would be advisable to delve deeper into the limitations of each of the patterns. It should be more critical.

**Discussion**

- This section seems too closely aligned with the project objectives. A more critical analysis is needed; otherwise, there is a risk of analytical bias. What would happen without funding? What would happen if an attempt were made to replicate the study? What aspects are most important to consider? What happens outside the contexts of the analysis?

**Conclusions**

- This section is very encouraging. It shows a positive bias. It needs to be more critical.

Is the case presented with sufficient detail to be useful for teaching or other practitioners?

Partly

Is the work clearly and accurately presented and does it cite the current literature?

Yes

If applicable, is the statistical analysis and its interpretation appropriate?

Not applicable

Are all the source data underlying the results available to ensure full reproducibility?

No source data required

Are the conclusions drawn adequately supported by the results?

Partly

Is the background of the case’s history and progression described in sufficient detail?

Partly

Reviewer Expertise:

Economy, innovation, technological change, digital agriculture

I confirm that I have read this submission and believe that I have an appropriate level of expertise to confirm that it is of an acceptable scientific standard, however I have significant reservations, as outlined above.

# Response

**Published:** 2026-02-13

**Authors:** Ferdinand Ferroli

**Affiliations:** Identity Valley Research, Germany

Thank you for the constructive feedback. The manuscript has been revised to improve clarity, balance, and critical reflection. **Abstract**

 The description of how pilot material was analysed has been clarified by stating the overall approach, data basis, and main analytical process more directly. **Introduction**

 The tone has been revised to be more neutral and analytical, with less project-affirming language. **Background (trade-off hacking and trade-off selection)**

 The conceptual discussion of trade-off hacking has been expanded, and clearer rationale has been added for focusing on the three main trade-offs in scope. **Pilot vignette section**

 A stronger critical dimension has been added through discussion of unresolved tensions and aspects that may remain challenging in practice. **Synthesis section**

 More explicit reflection has been included on the limitations and conditions of the identified patterns. **Discussion**

 The critical analysis has been deepened, including reflections on likely constraints in other implementation settings. **Conclusions**

 The conclusion has been revised to a more cautious framing, with clearer acknowledgement of limits and open questions.

# Reviewer report: Reviewer response for version 1

**DOI:** 10.21956/openreseurope.23550.r65045

**Published:** 2026-02-03

**Authors:** Charis M Galanakis

**Affiliations:** 1 Galanakis Laboratories, Chania, Greece

This study aims to reconceptualise ethical and functional trade-offs in data-driven food technologies through the lens of “trade-off hacking,” shifting from zero-sum compromises to innovation-oriented, user-centric solutions. By analysing eight Horizon Europe DRG4FOOD pilot projects, it identifies recurring tensions (privacy versus personalisation, performance versus explainability, and security versus user experience). It systematically examines how they are addressed in practice. The main outcome is a typology of reproducible design patterns that distinguishes technology-driven from governance-driven resolutions and demonstrates how trust, responsibility, and performance can be mutually reinforcing. The study is conceptually original, empirically grounded, and offers a compelling, well-argued contribution to responsible digital innovation and is highly commendable.

1. Clarify the methodological approach more explicitly, including case selection criteria, data sources, and analytical steps, to strengthen transparency and replicability and to help readers better assess the robustness and transferability of the findings across contexts.
2. Provide a more apparent justification for focusing on only three trade-offs, discussing whether other relevant tensions were considered or excluded, and how this choice may shape interpretations and limit the generalisability of conclusions in broader food systems.
3. Expand the discussion of limitations by more critically reflecting on potential biases introduced by the responsible incubator setting, funding incentives, and coaching support, and how these factors might affect applicability outside Horizon Europe programmes and markets.
4. Include more concrete comparative analysis across the pilot cases, possibly through a synthesis table or metrics, to better demonstrate the relative effectiveness of different strategies and avoid an overly descriptive, narrative-driven assessment of outcomes and impacts observed.
5. Add clearer implications for practitioners by translating identified patterns into actionable design guidance or decision criteria, helping developers understand when to prioritise technological solutions versus governance-driven approaches under real-world constraints, including scale, cost, and maturity levels.

Is the case presented with sufficient detail to be useful for teaching or other practitioners?

Yes

Is the work clearly and accurately presented and does it cite the current literature?

Yes

If applicable, is the statistical analysis and its interpretation appropriate?

Not applicable

Are all the source data underlying the results available to ensure full reproducibility?

Partly

Are the conclusions drawn adequately supported by the results?

Partly

Is the background of the case’s history and progression described in sufficient detail?

Yes

Reviewer Expertise:

food security; food waste

I confirm that I have read this submission and believe that I have an appropriate level of expertise to confirm that it is of an acceptable scientific standard, however I have significant reservations, as outlined above.

# Response

**Published:** 2026-02-13

**Authors:** Ferdinand Ferroli

**Affiliations:** Identity Valley Research, Germany

Thank you for the positive and helpful review. The manuscript has been revised in line with the main suggestions.

1. **Methodological transparency**
  The methodological approach is now presented more explicitly, including case selection, evidence sources, and analytical steps.
2. **Why three trade-offs**
  Clearer justification has been added for focusing on the three selected trade-offs, and the scope implications of this choice are now acknowledged more directly.
3. **Limitations and transferability**
  The limitations discussion has been expanded, with more explicit reflection on contextual influences and on transferability beyond similar programme environments.
4. **Cross-case comparison**
  Comparison remains qualitative in this version; no harmonised effectiveness metrics were introduced due to the exploratory design and heterogeneous evidence base. This is now stated as a limitation and identified as a priority for future research.
5. **Implications for practice**
  Practitioner-oriented guidance has been refined to make the identified patterns more actionable under real-world constraints.
